# Notes and News

**Published:** 1896-03-21

**Authors:** 


### Notes and News.

(Collected and Communicated.)

### HOSPITAL MEETINGS.

The Annual Meeting of the Poplar Hospital for Accidents will be held on Tuesday, 24th inst., at 5 p.m., at the hospital

The Anniversary Dinner of the West Ham Hospital will take place at the Holborn Restaurant on Wednesday, April 15th, at 7 p.m., Mr. A. F. Hills presiding.

The 46th Annual Meeting of the City of London and East London Dispensary will be held at the Dispensary, 35, Wilson Street, Finsbury, on Wednesday, March 25th, at 2.30 p.m.

H.R.H. the Duke of Cambridge will preside at a festival dinner in aid of the funds of the North London or University College Hospital, to be held in the King's Hall, Holborn Restaurant, on Wednesday, May 6th.

The Annual Festival Dinner in aid of King's College Hospital will be held at the Freemasons' Tavern, Great Queen Street, on Tuesday, April 14th, the Right Hon. Lord Russell of Killowen in the chair.

The Lord Mayor and his Sheriffs will visit the Royal Hospital for Children and Women on Friday, March 20th, at three o'clock.

An anonymous donor has given £40,000 for the purpose of erecting a hospital in Moscow for the treatment of medical cases.

The Goldsmiths' Company have made a grant of £200 in aid of the funds of the Metropolitan Hospital, Kingsland Road.

They have also given £250 to the Metropolitan Convalescent Institution, the office of which is at 32, Sackville Street, W.

Lord Wolverton has consented to preside at the festival dinner in aid of the funds of the City of London Hospital for Diseases of the Chest, Victoria Park.

It is proposed to establish a sanatorium for the West Coast of Africa as a memorial to the late Prince Henry of Battenberg. A locality has not yet been chosen.

Measles is unusually prevalent throughout the country, the death-rate being notably high.

A new site is to be presented to Stroud by Sir John Dorington, M.P., for the erection of a fever hospital, which is to take the place of the Oakridge Hospital, recently burnt by a mob, as reported in our columns.

Additions and alterations are to be made in connection with the Royal Hants Infirmary. The estimated sum required is £19,000. This amount will be met by the sale of invested funds, and by special subscriptions, which have now reached £5,200, leaving a considerable balance yet to be obtained.

His Royal Highness Prince Christian has consented to attend the festival dinner of the Royal Albert Orphan Asylum, Bagshot, on May 21st, presided over by the Duke of York. The Sheriffs of London will also attend.

An American contemporary acquaints us with a new habit said to be acquired by English ladies. This is the smoking of cigarettes made of tea. These pernicious articles are accused of engendering many nervous maladies. As yet we have failed to meet with a specimen of these cigarettes, or to hear of anyone who has either seen or used one.

It is hoped that the Duke and Duchess of York will open the new infirmary now in course of erecting at Halifax. The new institution will contain 140 beds, initially, with facilities to extend the accommodation to 300. The site is a good one, and the hospital is being built after the single-storey pavilion type.

The General Infirmary at Burton has again experienced great generosity from the Misses Ratcliff. Some time ago these ladies offered to present a ward for female patients to the institution at an estimated cost of £1,500. They now have expressed a desire to provide the whole of the front block at a further expenditure which will increase their donation to £2,500.

The Lord Mayor has kindly given permission for the annual meeting of the Factory Girls' Country Holiday Fund to be held at the Mansion House on Tuesday, April 28th, at three o'clock. The speakers will be Mrs. Fawcett, Lady Jeune, Mr. Asquith, Q.C., M.P., Colonel Barrington-Foote, the Rev. Dr. Welldon, and others. The fund is under the patronage of H.R.H. the Duchess of Teck. Tickets for the meeting can be had from the hon. secretary, Miss Canney, St. Peter's Rectory, Saffron Hill, London, E.C.

H.R.H. the Duchess of Connaught will receive purses of £5 5s. and upwards from children at the bazaar in aid of the North-Eastern Hospital for Children, at Queen's Hall, Langham Place, on June 23rd, after the opening ceremony. Collecting books (for purse collections) may be obtained from the secretary, Mr. T. Glenton-Kerr, 27, Clement's Lane, E.C. The hospital is much in need of help, and to place it in a satisfactory financial position the following sums are required this year: To pay off the debt, £3,000; to enable the hospital to pay its way this year, £2,500; increase in annual subscriptions required to give the hospital a fair chance of keeping out of debt for the future (477 subscriptions of £1 1s.). Six hundred and eighty-seven in-patients were received into the hospital in 1895 (of which number 174 were under two years of age); and out-patients numbered 15,519.

